# *Journal of Cannabis Research*: a new international, multi-disciplinary, open access journal

**DOI:** 10.1186/s42238-019-0005-x

**Published:** 2019-06-07

**Authors:** David A. Gorelick

**Affiliations:** University of Maryland School of Medicine, Baltimore, Maryland, USA

I am proud and excited to introduce a brand new scholarly journal, the *Journal of Cannabis Research*, sponsored by the Institute of Cannabis Research at Colorado State University - Pueblo and published with the assistance of Springer Nature, one of the world’s largest and most prominent academic publishers. One question immediately comes to mind: Why do we need this new journal now? I appreciate the opportunity to answer this question.

Academic inquiry about the cannabis plant, its products, and their effects has increased exponentially over the past several decades. This increase has occurred in all fields, from the plant biology and agriculture of the cannabis plant and the biomedical science underlying cannabinoids and the endocannabinoid system, to the health and public health effects of cannabis use and the regulation and socioeconomic impact of the cannabis industry (both legal and illegal). Up to now, the publications generated by this academic inquiry tend to be scattered across hundreds of journals in dozens of different academic fields. This dispersion makes it difficult for anyone to keep abreast of advances in more than a few aspects of cannabis science and to publish broadly multidisciplinary work that does not fit neatly into one topic.

Our new journal aims to overcome this limitation, with 10 topic sections that cover the entire range of cannabis-related science, as outlined above. Our editorial board includes more than 30 distinguished scientists and scholars from diverse fields. They work in 9 countries on 5 continents, so that the journal will encompass international points of view. The goal is not only to publish high-quality scholarly work, but also to foster and publish multi-disciplinary syntheses and cross-cultural analyses that will advance the field. To this end, the journal will publish reviews and commentaries, as well original research presenting newly generated data.

I believe that the 4 papers appearing in our inaugural issue well illustrate the breadth of our intended coverage. One paper (Schwabe and McGlaughlin, 2019) describes a study using micro-satellite genetic markers to evaluate the biological validity and consistency of cannabis strains that were initially classified on the basis of their marketing brand names. A second paper (Kumar et al., 2019) describes a detailed analysis of cannabis use patterns among a large convenience sample of 8345 US cannabis users who completed a web-based survey. A third paper (Peters and Foust, 2019) describes the association between the presence of legal recreational cannabis dispensaries in a community and high school students’ attitudes towards cannabis, based on a representative sample of 12 high schools in 7 communities in Colorado. The fourth paper (Yeung et al., 2019) describes a bibliometric analysis of the most highly cited papers describing biomedical studies of cannabinoids or the endocannabinoid system that were published between 1986 and 2016.

We look forward to your manuscript submissions and to your comments and suggestions, whether as reader or author (hopefully, both), for improving our journal in the future.
